# A Comparative Evaluation of the Estimation of Rabies Virus Antibodies among Free-Roaming, Vaccinated Dogs in Bengaluru, India

**DOI:** 10.3390/v14030484

**Published:** 2022-02-26

**Authors:** Lekshmi J. Das, Shrikrishna Isloor, Alur Kotrappa Santosh, Avinash Bhat, Ramakrishnaiah Sharada, Doddamane Rathnamma, Belamaranahally Muniveerappa Veeregowda, Konanduru Lingappa Phaniraj, Nageshkumar Abhijit Kumar, Abi T. Vanak

**Affiliations:** 1 KVAFSU-CVA Rabies Diagnostic Laboratory, Department of Microbiology, Veterinary College, KVAFSU, Bengaluru 560024, India; lekshmijdas10@gmail.com (L.J.D.); santoshakvet@yahoo.co.in (A.K.S.); sharadavet@gmail.com (R.S.); rathnarohit@gmail.com (D.R.); 2 Trouw Nutrition India Pvt. Ltd., Gachibowli, Hyderabad 500032, India; avinash.bhat.s@gmail.com; 3 OIE Reference Laboratory for Rabies, Department of Microbiology, Veterinary College, KVAFSU, Bengaluru 560024, India; drveeregowda@gmail.com (B.M.V.); drpaniraj@gmail.com (K.L.P.); 4 Ashoka Trust for Research in Ecology and the Environment, Jakkur, Bengaluru 560064, India; abhijit.kumar@atree.org (N.A.K.); avanak@atree.org (A.T.V.); 5 School of Life Sciences, University of KwaZulu-Natal, Durban 4041, South Africa; 6 DBT/Wellcome Trust India Alliance Program, Banjara Hills, Hyderabad 500034, India

**Keywords:** iELISA, rabies, serology, vaccination, zoonosis

## Abstract

Vaccination is the practical solution for the prevention of rabies in dogs. Assessment of the immunogenicity of vaccination includes estimation of specific rabies virus neutralizing antibodies (VNA) in the target species. We undertook a study to estimate the levels of VNA in free-roaming dogs with a history of rabies vaccination in Bengaluru city, India. We compared the rapid fluorescent focus inhibition test (RFFIT) and an in-house quantitative indirect ELISA (iELISA). The study area comprised the jurisdiction of Bruhat Bengaluru Mahanagara Palike (BBMP), the Bengaluru civic body. The BBMP, along with several non-government organizations (NGO), were conducting a trap- neuter- vaccinate- release program for the prevention of dog rabies. Serum samples were collected from 250 free-roaming dogs from representative regions of BBMP, of which 125 had a VNA titre of 0.5 IU or more by the RFFIT. Furthermore, 126 dogs showed percent positivity values (PP values) more than the cut off PP value of 57.1 by the iELISA, accounting for 50.4% of satisfactory post-vaccinal serum conversion. The sensitivity and specificity of the iELISA was 94.4% and 95.2%, respectively. Based on these data, a quantitative iELISA may be a complementary tool for sero-monitoring immune responses of free-ranging animals after rabies vaccination.

## 1. Introduction

Rabies is a zoonotic viral disease caused by RNA viruses in the genus Lyssavirus of the family *Rhabdoviridae* [1]. Considering its agricultural and public health significance, rabies virus (RABV) is the most important member of the genus. The disease is endemic in most African and Asian countries and leads to a fatal encephalomyelitis once the signs appear. Globally, rabies kills tens of thousands of people annually, and most (99%) cases are transmitted by domestic dogs [2,3,4]. India has the highest rabies burden, and a contributing factor is the existence of a dog population of approximately 1.7 million [5], with an estimated dog: human, ratio of 1:36 [6]. Dogs fall into four broad categories: pets (restricted, supervised); family dogs (partially restricted, wholly dependent); community dogs (unrestricted, partially dependent); and feral dogs (unrestricted, independent). Most dogs in India are thought to fall into the last three categories [7]. This feature is a major hurdle in rabies prevention and control. Dogs that are not confined to an owner’s property are considered free-roaming [8]. These free-roaming dogs could be both owned and allowed to roam freely or stray (including recently owned but lost from home or abandoned). Strays may also include quasi-owned animals that are cared for or considered to belong to a neighborhood. The term free-roaming simply describes a lack of confinement [9,10].

Since dogs play a major role in RABV transmission to humans, prevention of human rabies mediated by dogs is dependent upon a combination of modern human rabies postexposure prophylaxis (PEP) and mass preexposure vaccination of the animal reservoirs. Properly conducted and coordinated mass vaccination campaigns of dogs should prevent most human exposures to RABV. Approximately 70% of a dog population should be immune to rabies in order to attain an epizootiological baseline of herd immunity in a population [3]. The induction of rabies virus neutralizing antibodies (VNA), directed against the viral glycoprotein is a key component in vaccine response [11]. A successful rabies vaccination should result in the rapid development of VNA, which can be assessed using either the rapid fluorescent focus inhibition test (RFFIT) or the fluorescent antibody virus neutralization test (FAVN) test [12,13,14]. A serum VNA titre of at least 0.5 IU/mL is considered as indicative of an adequate immunological response to vaccination [3].

In India, the trap-neuter-vaccinate-release (TNVR) program for stray dog vaccination is not monitored for sero-conversion by analyzing rabies VNA. Bengaluru city has been in the forefront of rabies vaccination activities as its medical and veterinary organizations contribute significantly for rabies management (and considering that dog bite cases here are comparatively high). We provide an assessment of a current rabies vaccination program in the city at the ground level through a determination of VNA in a sample of vaccinated dogs using the RFFIT. However, the RFFIT is time consuming, expensive, and requires highly trained laboratory staff and live virus handling. Developing countries such as India need alternative, safe, rapid, economical, and user-friendly methods due to an inadequate number of sophisticated laboratories.

In light of this, this study assessed rabies VNA in a sample of free-roaming vaccinated dogs using the RFFIT and compared the results with a quantitative, indirect ELISA (iELISA). Nevertheless, ELISA detects binding antibodies to viral antigens and RFFIT assays in-vitro virus neutralizing antibodies.

## 2. Materials and Methods

### 2.1. Study Area

**Figure 1:**
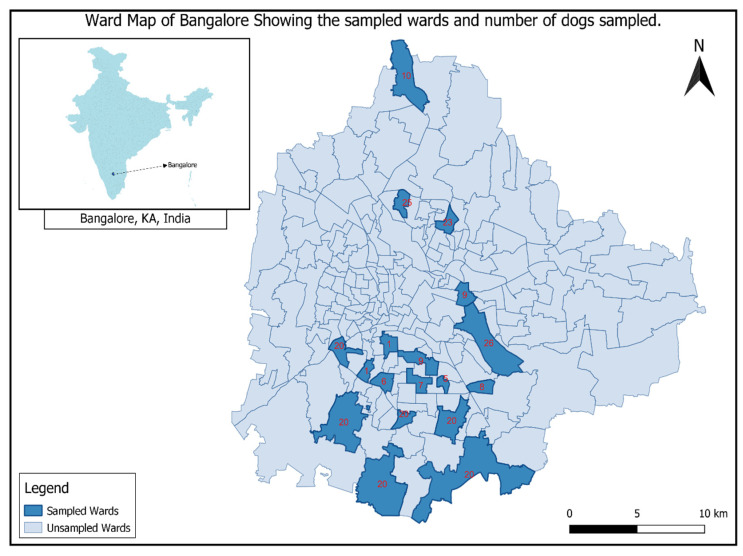
Map of Bengaluru city, showing the distribution of various wards of north and south Bengaluru with number of samples collected from each ward.

We used a convenience sampling method to select 18 different municipal wards in Bengaluru city in Karnataka state in southern India. Bengaluru is the second largest city in southern India, with a human population of 8 million, and a free-ranging dog population of more than 300,000 [15]. We accompanied teams from animal welfare organizations who were undertaking routine anti-rabies vaccination programs in Bengaluru. A total of 250 blood samples were collected from free-roaming dogs between January and June 2018 from 18 different wards of North and South Bangalore (Figure 1). All of these dogs were vaccinated by the animal welfare organizations in the period of 2017–2018 with the Raksharab vaccine supplied by BBMP.

### 2.2. Serum Sample Collection

Free-roaming dogs, with notched ears, indicating that they have undergone the animal birth control (ABC) procedure with simultaneous anti-rabies vaccination (ARV), were humanely captured by trained dog catchers either using nets or by hand for sample collection [16]. While collecting blood samples, gender, geo-tagged photos of dogs, color, body condition score, and the unique code were recorded using a custom web application developed at the Ashoka Trust for Research in Ecology and Environment (ATREE), Bengaluru for their population dynamics study. Field and laboratory protocols were approved by the Institutional Animal Ethics Committee (IAEC Number: VCH/IAEC/2018/62, dated 13 March 2018), and according to the guidelines of the Committee for the Purpose of Control and Supervision of Experiments on Animals (CPCSEA), Government of India. Blood samples (2–3 mL) were collected into serum vacutainers (BD Vacutainer^®^ SSTTM II Advance tubes, BD Diagnostics, Oxford Science Park, Oxford OX4 4DQ. mfg. date: December 2017) and left to clot at room temperature for 2–3 h and then centrifuged at 4000 g for 3 min. The separated serum samples were preserved at −20 °C.

### 2.3. Virus and BHK-21 Cells

BHK-21 cells were propagated in Dulbecco’s Modified Eagle’s Medium (DMEM) with 10% fetal bovine serum (FBS) and antibiotics, and the titrated PV 3462 strain of RABV (Dr. Oscar Larghi’s strain) obtained from the Pasteur Institute, Conoor, was used [17].

### 2.4. Rapid Fluorescent Focus Inhibition Test

The RFFIT procedure, standardized by Neelufer, 2016 [18], was used on all 250 serum samples in the study. Serum samples were incubated in a water bath at 56 °C for 30 min. for complement inactivation [19,20]. The samples were serially diluted in a flat-bottomed 96-well microtitre plate (Nunc MaxiSorp™ flat-bottom, Thermo Fisher Scientific, Waltham, MA, USA). 100 TCID_50_ of virus were added to all wells except cell controls and incubated at 37 °C for 90 min. Further, approximately 30,000 BHK-21 cells/well were seeded and incubated at 37 °C for 48 h in a 5% CO_2_ incubator. The medium was decanted from the plate without disturbing the monolayer and the cells were fixed by incubating in 70% chilled acetone for 30 min at −20 °C. The fixed cells were incubated with a 1:20 diluted fluorescein-labeled anti-RABV nucleoprotein antibody (Fujirebio Diagnostics, Devault, Malvern, PA, USA) for 1 h at 37 °C. The plates were examined using a fluorescent microscope, and fluorescent foci were counted. The titre of rabies VNA were estimated in comparison with WHO reference serum. The highest dilution of the serum at which the complete neutralization of RABV was considered for estimation of the VNA titre. For the purpose of this study, we considered a VNA titre of 0.5 IU/mL serum as adequate. The titre was estimated based on the following equation.

$$\mathrm{Antibody}\;\mathrm{Titre}=\frac{\mathrm{Reciprocal}\;\mathrm{of}\;\mathrm{end}\;\mathrm{point}\;\mathrm{titre}\;\mathrm{of}\;\mathrm{sample}\;\times \;\mathrm{Titre}\;\mathrm{of}\;\mathrm{reference}\;\mathrm{serum}\;\left(\mathrm{IU}\right)}{\mathrm{Reciprocal}\;\mathrm{of}\;\mathrm{end}\;\mathrm{point}\;\mathrm{titre}\;\mathrm{of}\;\mathrm{reference}\;\mathrm{serum}}$$

### 2.5. Indirect Enzyme-Linked Immunosorbant Assay

The iELISA using a baculovirus-expressed RABV glycoprotein was employed on the 250 canine serum samples [21]. Antigen at 500 ng/100 μL/well was coated by incubating at 4 °C overnight. The contents of the wells were discarded, and the plates were washed two times with phosphate-buffered saline (PBS), pH 7.2. Blocking was carried out using 4% skim milk powder for 1 h. After twice washing with PBS, diluted serum samples were added and the plates were incubated at 37 °C for 120 min. The controls included serially diluted positive, negative, and conjugate controls and blank wells. Afterwards, 100 μL of 1:15,000 diluted rabbit anti-canine IgG HRP conjugate (Sigma-Aldrich, St. Louis, Missouri, USA) were added to each well and incubated at 37 °C for 60 min. Chromogen-substrate (OPD-H_2_O_2_) was added after washing and finally, 2.5 N HCl was added to stop the reaction. Absorbance values were read at 492 nm using an ELISA reader.

### 2.6. Statistical Analysis

The effectiveness of iELISA was evaluated using two tailed Spearman correlation analysis by comparing it with RFFIT. The strength of iELISA with respect to RFFIT was evaluated using Cohen’s kappa statistics. All statistical analysis and regression fittings were performed using GraphPad Prism Version 5.0 and 6.0 for Windows, GraphPad Software, San Diego, CA, USA (www.graphpad.com, accessed on 22 January 2022).

## 3. Results

In this study, 250 serum samples from vaccinated dogs were collected from north (*n* = 97) and south (*n* = 153) parts of Bengaluru and analyzed by the RFFIT. In the assay, fluorescent foci was observed in the virus control (100 TCID50 virus) as well as in the serum samples with inadequate rabies VNA indicating virus replication. Serum samples containing rabies VNA antibodies (defined in our study to contain at least 0.5 IU/mL) displayed complete neutralization against RABV.

**Figure 2:**
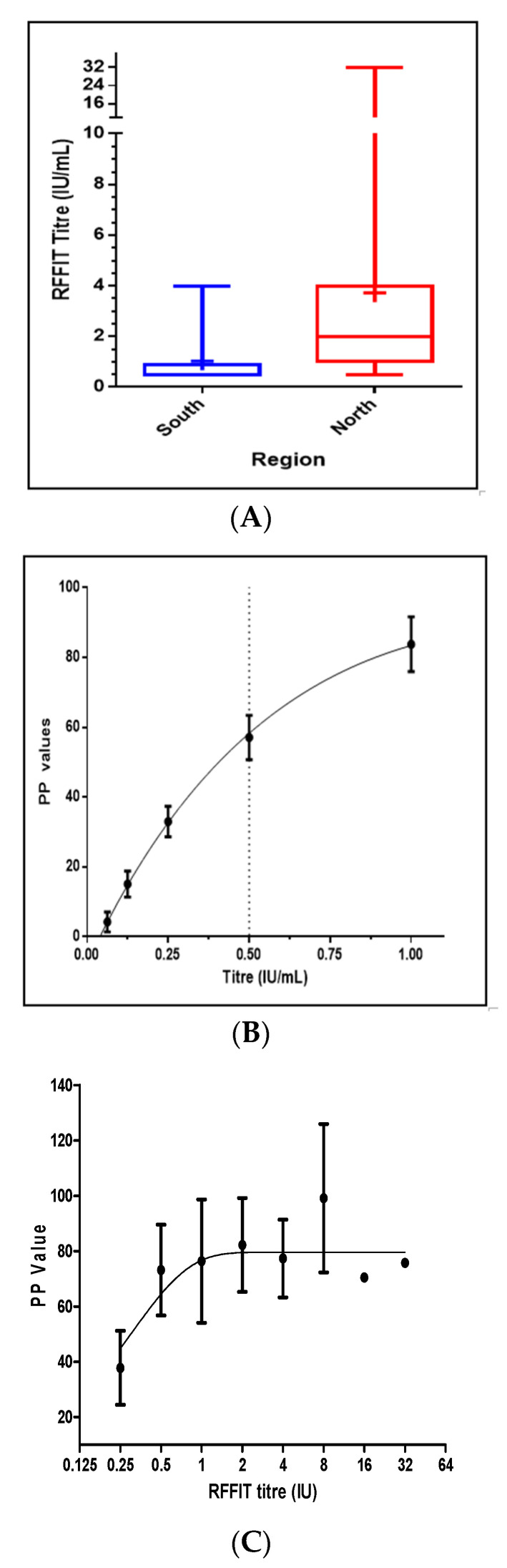
Tukey Box and Whisker Plot for region-wise rabies virus neutralizing antibody (VNA) titres in vaccinated dogs (**A**) and graph showing rabies VNA titre versus percent positivity (PP) values of control sera (**B**). Nonlinear regression plot depicting the percent positivity (PP) values with standard deviation of tested samples against the corresponding RFFIT VNA titre (**C**).

In comparison, 66.0% and 40.6% of dogs from north and south Bengaluru, respectively had VNA ≥ 0.5 IU. Of the 250 samples analyzed by the RFFIT, 125 (50%) had rabies VNA titres ≥ 0.5 IU. The Tukey box and Whisker diagrams to show mean, median, and distribution of inter-quartile range are depicted in Figure 2A. The highest rabies VNA titres in the North and the South zone were 32 and 4 IU/mL, respectively.

The 250 samples were also subjected to an iELISA, standardized previously as described [21]. The average OD values converted to PP values of serum control were plotted against corresponding VNA titres and a graph, which indicated the percent positivity(pp) value of 57.09 corresponded to VNA titre of 0.5 IU/mL, was generated (Figure 2B). The iELISA for 250 test serum samples showed a Spearman correlation coefficient r of 0.22 with *p* value 0.0306, indicating a significant correlation (*p* <0.05), as shown (Figure 2C) against the RFFIT. In the iELISA, of 250 serum samples, 126 (50.4%) were found to contain an adequate level of antibodies against rabies. The sensitivity and specificity of the iELISA were 94.4%, 95.2%, respectively, and the kappa value was 0.89 indicating very good agreement with RFFIT results.

## 4. Discussions

Dog-bites are a huge financial burden in India. Dog bite victims experience a prolonged anxiety if the biting dog is free-roaming due to the high prevalence of rabies. Prevention of human rabies primarily depends on human PEP and the management of dog rabies, which can be achieved only by mass vaccination. These mass vaccination campaigns should be monitored scientifically. This study assessed rabies VNA levels in a rabies vaccination program in Bengaluru city, India.

This present study results revealed that 50% of sampled dogs showed an adequate rabies VNA titre (i.e., >0.5 IU), with seroconversion rates varying (from 40.5% to 66.0%) between the north and south zones. The WHO recommends 70% as an epizootiological baseline of herd immunity in dogs [3]. The stated 70% vaccination coverage refers to the percentage of dogs protected against a rabies infection. Although in the present study (based on serology) this proportion was not reached, cell mediated immune response can still have protective immunity at lower titres due to shift from circulating antibodies to a memory cell-based immunity. The lower level of rabies VNA may be due to a combination of host, vaccine, and methodological factors, including individual variation, time since vaccination, cold chain issues, a lack of stable vaccination programs, individual identification systems, and vaccination failure.

An earlier study from Chandigarh, India, observed that only 1% of street dogs had adequate titres [22], as evaluated by using a commercially available ELISA. Another sero-surveillance study using an iELISA from Mumbai showed an overall low seroprevalence of anti-RABV antibody in stray dogs at the level of 39.2% [23] and a study from Chennai revealed an inadequate titre in 60% of vaccinated dogs [24]. Similar results of inadequate levels (i.e., 62%) have been reported in stray dogs in Bangkok, where a commercial ELISA was used [25]. Another study using a commercial ELISA in free- roaming and stray dogs in Ilorin, Nigeria, found that only 42.6% of dogs had an adequate antibody titre [26], with confined dogs showed better seroconversion (49.1%) than free-roaming (37.7%) and stray (7.7%) dogs.

Currently available tests for assessing rabies VNA are the RFFIT and FAVN. Since both tests are expensive, time consuming, and require live virus handling, other tests such as an iELISA can be adapted (although VNA are not directly evaluated). In this study, a recombinant RABV glycoprotein based in-house ELISA was used, as developed previously [21]. This was evaluated by comparing the test with the standard RFFIT. Diagnostic parameters and statistical analyses showed a high agreement between the results of iELISA and RFFIT. A similar observation has been made by other researchers for ELISA with post-vaccinal human sera [27] and random samples from different breeds in India [28], as well as for human and murine sera [29,30,31]. No significant difference was also reported while investigating two commercially available anti-rabies vaccines in a five-dose vaccination regimen in apparently healthy street dogs [32]. Alternatively, correlation between the ELISA and RFFIT was noted in experimental but not in field conditions. It was inferred that this may be due to the poly-parasitism and malnutrition which may depress immune response and affect data interpretation [33]. Nevertheless, these studies suggest that the iELISA can be complementary to RFFIT/ FAVN, especially in monitoring the mass immunization programs, and can be further standardized with intermittent verification. The seroconversion level against RABV antigens among free-roaming vaccinated dogs in the Bengaluru city Municipal Corporation limit was one method of monitoring. Given that we only sampled dogs with a known history of vaccination, in certain pockets of north and south Bengaluru, and recognizing that not all dogs are captured for vaccination, the actual level of seropositive animals may be different than we report. A coordinated public health and veterinary services program with a robust system of annual vaccination is needed. The outcome of the present study indicated that a quantitative ELISA is user friendly, rapid, and can be carried out in less sophisticated laboratories. However, the use of tools to evaluate serological response is only one aspect for monitoring the utility of a program. We also recommend the need for enhanced rabies surveillance for detection of rabies occurrence as a part of the National Action Plan for Rabies Elimination (NAPRE) [34].

## 5. Conclusions

This study compared the rabies VNA response after vaccination programs in free roaming dogs in Bengaluru, India. A 50% seroconversion was observed. Furthermore, agreement was found between the serosurvey techniques using the RFFIT and iELISA. The iELISA may be adopted for further largescale sero-monitoring studies, since the RFFIT is a time consuming and complicated test for many investigators within developing countries such as India.

## Data Availability

Data are available upon request.

## References

[1] ICTV Virus Taxonomy: 2020 Release. https://talk.ictvonline.org/taxonomy.

[2] Hampson K., Coudeville L., Lembo T., Sambo M., Kieffer A., Attlan M., Barrat J., Blanton J.D., Briggs D.J., Cleaveland S. (2015). Estimating the global burden of endemic canine rabies. PLoS Negl. Trop. Dis..

[3] World Health Organization (2018). WHO Expert Consultation on Rabies.

[4] Gompper M.E. (2013). Free-Ranging Dogs and Wildlife Conservation.

[5] Sudarshan M.K., Madhusudan S.N., Mahendra B.J., Rao N.S.N., Ashwath Narayana D.H., Abdul Rahman S. (2007). Assessing the burden of human rabies in India: Results of a national multi-center epidemiological survey. Int. J. Infect. Dis..

[6] Sudarshan M.K., Mahendra B.J., Madhusudana S.N., Narayana D.A., Rahman A., Rao N.S.N., X-Meslin F., Lobo D., Ravikumar K. (2006). An epidemiological study of animal bites in India: Results of a WHO sponsored national multi-centric rabies survey. J. Commun. Dis..

[7] Hiby E.F., Hiby L.R., Serpell J. (2017). Dog population management. The Domestic Dog. Its Evolution, Behaviour and Interactions with People.

[8] Vanak A.T., Gompper M.E. (2009). Dogs *Canis familiaris* as carnivores: Their role and function in intraguild competition. Mamm. Rev..

[9] Smith L.M., Hartmann S., Munteanu A.M., Dalla Villa P., Quinnell R.J., Collins L.M. (2019). The effectiveness of dog population management: A systematic review. Animals.

[10] Patronek G.J. (1998). Free-roaming and feral cats—their impact on wildlife and human beings. J. Am. Vet. Med. Assoc..

[11] Katz I.S.S., Guedes F., Fernandes E.R., dos Ramos Silva S. (2017). Immunological aspects of rabies: A literature review. Arch. Virol..

[12] Cliquet F., Aubert M., Sagne L. (1998). Development of a fluorescent antibody virus neutralisation test (FAVN test) for the quantitation of rabies-neutralising antibody. J. Immunol. Methods.

[13] Rupprecht C.E., Fooks A.R., Abela-Ridder B., World Health Organization (2018). Laboratory Techniques in Rabies.

[14] Timiryasova T.M., Luo P., Zheng L., Singer A., Zedar R., Garg S., Petit C., Moore S., Hu B.T., Brown M. (2019). Rapid fluorescent focus inhibition test optimization and validation: Improved detection of neutralizing antibodies to rabies virus. J. Immunol. Methods.

[15] Animal Welfare Board of India (2001). Animal Birth Control (Dogs) Rules. http://www.awbi.in/policy_acts_rules.html.

[16] Belsare A., Vanak A.T. (2020). Modelling the challenges of managing free-ranging dog populations. Sci. Rep..

[17] Santosh A.K., Isloor S., Rathnamma D., Sharada R., Sunilkumar K.M., Balamurugan V., Yathiraj S., Satyanarayana M.L. (2017). Assessment of Humoral Immune Response in Vaccinated Domestic Dogs and Cats Intended for Pet Travel from India by Rapid Florescent Focus Inhibition Test (RFFIT). https://krishi.icar.gov.in..

[18] Neelufer M.S. (2016). Standardisation and Application of Rabies Virus Neutralizing Antibody Assay for Assessment of Vaccinal Efficacy in Dogs. Master’s Thesis.

[19] Moore S.M., Hanlon C.A. (2010). Rabies-specific antibodies: Measuring surrogates of protection against a fatal disease. PloS. Negl. Trop. Dis..

[20] Kramer B., Schildger H., Nicol H. (2009). The rapid fluorescent focus inhibition test (RFFIT) is a suitable method for batch potency testing of inactivated rabies vaccines. Biologicals.

[21] Santosh A.K. (2017). Development of ELISA and its Comparative Evaluation with RFFIT for Estimation of Anti-Rabies Vaccinal Antibodies in Dogs. Ph.D. Thesis.

[22] Singh M.P., Goyal K., Majumdar M., Ratho R.K. (2011). Prevalence of rabies antibodies in street and household dogs. Trop. Anim. Health Prod..

[23] Nale J.M., Pharande R.R., Majee S.B., Gandge R.S., Sawane M.P., Ingle S.A. (2021). Serosurveillance of rabies antibodies in dogs in Mumbai region by using indirect ELISA. Comp. Immunol. Microbiol. Infect. Dis..

[24] Yale G., Sudarshan S., Taj S., Patchimuthu G.I., Mangalanathan B.V., Belludi A.Y., Shampur M.N., Krishnaswamy T.G., Mazeri S. (2021). Investigation of protective level of rabies antibodies in vaccinated dogs in Chennai, India. Vet. Rec. Open.

[25] Kasempimolporn S., Sichanasai B., Saengseesom W., Puempumpanich S., Chatraporn S., Sitprija V. (2007). Prevalence of rabies virus infection and rabies antibody in stray dogs: A survey in Bangkok, Thailand. Prev. Vet. Med..

[26] Olugasa O.O., Aiyedun J.O., Emikpe B.O. (2011). Prevalence of antibody against rabies among confined, free roaming and stray dogs in a transit city of Nigeria. Vet. Ital..

[27] Muhamuda K., Madhusudana S.N., Ravi V. (2007). Development and evaluation of a competitive ELISA for estimation of rabies neutralizing antibodies after post-exposure rabies vaccination in humans. Int. J. Infect. Dis..

[28] Shyamsundar K.A., Isloor S., Madhusudhana S.N., Mahesh V., Yathiraj S., Nandini V.M., Rathanamma D., Veeregowda B.M., Satyanarayana M.L., Bhat M.N. Comparative evaluation of RFFIT and RABV G-protein based ELISA for seromonitoring of anti-rabies vaccinal antibodies in domestic dogs in and around Bengaluru. Proceedings of the 16th National Conference of APCRICON.

[29] Feyssaguet M., Dacheux L., Audry L., Compoint A., Morize J.L., Blanchard I., Bourhy H. (2007). Multicenter comparative study of a new ELISA, PLATELIA^TM^ RABIES II, for the detection and titration of anti-rabies glycoprotein antibodies and comparison with the rapid fluorescent focus inhibition test (RFFIT) on human samples from vaccinated and non-vaccinated people. Vaccine.

[30] Zhao R., Yu P., Shan Y., Thirumeni N., Li M., Lv Y., Li J., Ren W., Huang L., Wei J. (2019). Rabies virus glycoprotein serology ELISA for measurement of neutralizing antibodies in sera of vaccinated human subjects. Vaccine.

[31] Debnath A., Pathak D.C., Narayan Ramamurthy G.M., Pandey A.B., Upmanyu V., Tiwari A.K., Saravanan R., Chellappa M.M., Dey S. (2019). Serological profiling of rabies antibodies by enzyme-linked immunosorbent assay and its comparative analysis with rapid fluorescent focus inhibition test in mouse model. Vet. World..

[32] Manickam R., Basheera M.D., Jayakumar R. (2008). Post-exposure prophylaxis (PEP) of rabies-infected Indian street dogs. Vaccine.

[33] Bahloul C., Taieb D., Kaabi B., Diouani M.F., Hadjahmed S.B., Chtourou Y., B’chir B.I., Dellagi K. (2005). Comparative evaluation of specific ELISA and RFFIT antibody assays in the assessment of dog immunity against rabies. Epidemiol. Infect..

[34] National Action Plan for Eliminating Dog Mediated Rabies from India. https://ncdc.gov.in/WriteReaData/l892s/25879243771600146411.pdf.

